# Pre-exposure Schedule Effects on Generalization of Taste Aversion and Palatability for Thirsty and Not Thirsty Rats

**DOI:** 10.3389/fpsyg.2018.00878

**Published:** 2018-06-05

**Authors:** Rocío Angulo

**Affiliations:** Universidad Autónoma de Chile, Santiago, Chile

**Keywords:** perceptual learning, pavlovian conditioning, deprivation, discrimination, generalization, palatability, taste aversion, rats

## Abstract

The study reported four experiments aiming to test the effects of the pre-exposure schedule and water deprivation on the generalization of a conditioned taste aversion in rats, with a particular focus on testing whether or not the concurrent schedule might enhance generalization. In two experiments, non-water-deprived rats received concurrent, intermixed, or blocked exposure to a sweet-acid solution and a salty-acid solution before conditioning of one of these compounds and testing of both flavors. During pre-exposure, the rats consumed a greater amount of the sweet-acid solution than the salty-acid solution (Experiments 1 and 2), consumption of the former increasing during pre-exposure while consumption of the latter decreased (Experiment 1). Furthermore, consumption of the salty-acid solution was lower during concurrent than intermixed or blocked pre-exposure (Experiment 1 and 2) while consumption of the sweet-acid solution was greater during intermixed than concurrent or blocked pre-exposure (Experiment 1). It is discussed whether the pre-exposure schedule might modify stimulus perception beyond the mere enhancement of stimulus differentiation, by, for instance, affecting the palatability of gustatory stimuli. Evidence for enhanced generalization after concurrent pre-exposure was not found for either deprived (Experiments 1, 2, and 3) or non-deprived rats (Experiments 3 and 4), with deprivation leading to a general increase in consumption of both the conditioned and test flavors. This then raised the question of whether or not concurrent pre-exposure to flavors always increases generalization between them. The present study highlights the importance of this issue for various accounts of perceptual learning.

## Introduction

A conditioned taste aversion established to one stimulus (e.g., AX) will, to some degree, generalize to another similar stimulus (e.g., BX), depending on the schedule with which the stimuli have been presented previously (e.g., Honey and Hall, 1989; Mackintosh et al., 1991). In particular, generalization between the stimuli appears to be lower after intermixed (i.e., AX, BX, AX, BX…, the stimuli being presented few hours apart) than blocked (i.e., AX, AX,…, BX, BX…) pre-exposure (e.g., Symonds and Hall, 1995, 1997; Mondragón and Hall, 2002). But shorter intervals (a few minutes or seconds) between intermixed presentations of the stimuli (e.g., Bennett and Mackintosh, 1999; Wills and Mackintosh, 1999) or concurrent pre-exposure (e.g., Alonso and Hall, 1999; Rodríguez and Alonso, 2008), seems to increase generalization with respect to the standard intermixed or blocked schedules.

Within the field of perceptual learning, it is widely accepted that generalization decrements after stimulus pre-exposure must rely, at least in part, on changes in the extent to which the stimuli can be differentiated as a consequence of stimulus pre-exposure (see Hall, 1991; Mitchell and Hall, 2014, for a review). To the extent that less generalization would be expected between stimuli that are better differentiated from each other, then such an effect should be more marked when using an intermixed pre-exposure schedule, compared to a schedule in which the stimuli are pre-exposed in a series of blocks. According to such an analysis, it is expected that stimulus differentiation would be poorer after rapid-intermixed or concurrent pre-exposure relative to standard intermixed or blocked pre-exposure. A number of human perceptual learning experiments, however, have found quite the opposite result. In particular, several studies have established that people are more accurate in judging two similar visual stimuli as different, or assigning such stimuli to different artificial categories following concurrent exposure in comparison with either intermixed or blocked schedules (e.g., Mundy et al., 2007, 2009; Angulo and Alonso, 2012, 2013). It has therefore been concluded that at least for humans, the concurrent schedule increases stimulus differentiation to a greater extent than the intermixed or blocked schedules. This apparent discrepancy between the results of studies conducted with human and non-human animals has been explained in several ways.

For example, it has been argued that, even in the case in which concurrent pre-exposure is able to enhance stimulus differentiation for non-human animals in the way described for people, various sources of generalization might still be enhanced by this schedule. According to some authors, concurrent pre-exposure to the stimuli will strengthen the associative excitatory links between them (e.g., Honey et al., 1994; Alonso and Hall, 1999; Rodríguez et al., 2008) and, such links would be able to increase generalization between the stimuli, even in the case in which the subjects are able to discriminate between them. Indeed, it is expected that excitatory links would be established between concurrently presented stimuli only in the case in which such stimuli were discriminable. It has also been suggested that common elements of the stimuli (X, in the example outlined above) could be more strongly conditioned after concurrent than intermixed or blocked pre-exposure (e.g., Bennett and Mackintosh, 1999; Rodríguez and Alonso, 2008). Thus, irrespective of how well animals may differentiate between the stimuli after concurrent pre-exposure, the size of the generalized conditioned response (CR) will be stronger, simply because learning acquired by the common elements would be greater.

Other analyses have focused on the extent to which the instructions given to participants during the pre-exposure phase could play a role in determining the outcome of human perceptual learning studies. To date, human studies assessing the concurrent schedule (e.g., Mundy et al., 2007, 2009; Angulo and Alonso, 2012, 2013) have specifically asked participants to look for differences between the stimuli during pre-exposure, while for other animals, stimuli are assumed to be “merely” pre-exposed (e.g., Hall, 1991, 2001). It has been proposed that, in humans, instructions may activate top-down strategies for processing the stimuli that would not be operating in situations in which mere exposure is given to other animals (e.g., Mitchell and Hall, 2014). According to such analyses, the greatest benefit for humans (although not for other animals) of the concurrent schedule may lie in an interaction of some sort between the pre-exposure schedule and the instructions asking the participant to seek out stimulus differences (see Nelson, 2009; Mitchell and Hall, 2014, for a review of this discussion). A recent study conducted with humans found that, in the absence of instructions asking participants to specifically look for stimulus differences, people were less accurate in judging the pre-exposed stimuli as being same or different, following concurrent pre-exposure (Angulo et al., unpublished). It might therefore be tempting to conclude that the effects of the pre-exposure schedule could vary according to the specific demands of the instructions given during pre-exposure and, if this were indeed the case for humans, one might wonder whether the same may also be true for other animals. Rats and other non-human animals cannot be verbally instructed during pre-exposure. But it could still be the case that the animals’ environment (external or internal) during pre-exposure may dictate the way in which the stimuli are processed, in the same way that verbal instructions are thought to influence stimulus processing in humans.

Experimental procedures employing taste aversion preparations with rats usually begin with a regime of water deprivation that is maintained throughout the experiment (e.g., Symonds and Hall, 1995, 1997; Honey and Hall, 1989; Mackintosh et al., 1991; Rodríguez and Alonso, 2008). Thus, the procedure induces a state of fluid deprivation under which the critical flavored solutions will be presented. In such a situation, rats receive exposure to stimuli with motivational value in a state of fluid depletion. It might therefore be a matter of debate as to what extent the rats are receiving mere exposure to neutral stimuli, as has traditionally been assumed. It might be possible to argue that the state of water deprivation — a part of the animal’s internal environment — might provide some “like-instructions” for processing the stimuli. Accordingly, the aim of the present study was to examine the impact of giving the animals free access to water during the pre-exposure phase (i.e., not inducing a state of water deprivation). In particular, we wanted to explore whether this change in the deprivation state would allow us to observe differences in the extent to which the schedule of pre-exposure has an effect on stimulus generalization, using a standard taste aversion procedure. Because discrepancies between the studies conducted with human and non-human animals arise from the effects of the concurrent pre-exposure schedule, we focused particularly on the effects of this schedule.

## Experiment 1

Using a conditioned taste aversion procedure, Experiment 1 tested the effect of concurrent, intermixed, and blocked pre-exposure to two similar flavored compound solutions, AX and BX, on subsequent generalization between them. Whilst this issue has previously been addressed (Rodríguez and Alonso, 2008; Rodríguez et al., 2008), the rats were fluid deprived during pre-exposure (but see Alonso and Hall, 1999 for concurrent and blocked pre-exposure effects when the flavored compounds were presented *ad libitum*). In this experiment, however, the deprivation regime began after pre-exposure. The experiment included eight groups of rats. Half of these groups received a single taste aversion conditioning trial with the compound AX (paired groups, - P) after pre-exposure, while the other half received presentation of the stimulus AX and the induced illness separately (unpaired groups, - U). Within the paired and unpaired groups, one received a concurrent pre-exposure schedule (e.g., AX–BX, AX–BX, AX–BX, etc., Groups CNC-P and CNC-U), another received the intermixed schedule (e.g., AX, BX, AX, BX, etc., Groups INT-P and INT-U) and the third the blocked schedule (e.g., AX, AX, etc., BX, BX, etc., Groups BLK-P and BLK-U). The remaining two groups received no pre-exposure to AX and BX before the induced illness (Groups CTRL-P and CTRL-U). Subsequently, all the rats received a single consumption test with the AX flavor, and another test with BX. Generalization between these stimuli was then assessed in two ways: by comparing AX and BX consumption in the paired groups; and by comparing consumption of BX in the paired groups with consumption of BX in the unpaired groups. Different consumption levels of AX and BX in the paired groups would indicate discrimination between the two stimuli. Since AX but not BX was aversively conditioned, lower consumption of AX than BX would be expected if rats were indeed able to differentiate between the stimuli. No differences between consumption of AX and BX in the paired groups would, however, be taken to indicate generalization between the stimuli. On the other hand, lower consumption of BX in the paired than in their respective unpaired control groups would also be indicative of generalization. Since no stimulus was conditioned in the unpaired groups, and thus there would be no aversion to be generalized from one stimulus to the other, consumption of BX and AX should be similar in this case, with consumption of BX being of the level that would be expected in the case of “zero generalization.”

Experiment 1 also measured the free consumption of the two flavored solutions employed as stimuli, AX and BX, during concurrent, intermixed, and blocked pre-exposure. The reason for this is that it has recently been suggested (Mitchell and Hall, 2014), though not empirically tested, that rats might be able to differentiate *a priori* the stimuli usually employed in perceptual learning studies. For the current theoretical accounts of perceptual learning (e.g., McLaren and Mackintosh, 2000; Hall, 2003; Mitchell et al., 2008), it becomes crucial to test this possibility. Such accounts have been developed primarily within the framework of animal research and have provided evidence for mechanisms that are able to explain stimulus differentiation as a consequence of pre-exposure. But, if the stimuli are differentiated *a priori*, then differentiation must clearly not be a consequence of such proposed mechanisms. As in previous studies (e.g., Mackintosh et al., 1991; Symonds and Hall, 1995), the two flavored compound solutions used here as stimuli shared a common acid flavor (X), and were distinguished by the addition of other distinctive flavors (A and B), which are sugar and salt (counterbalanced in each group, see below). The sweet solution was expected to be more palatable than the salty solution. Thus, if rats were able to differentiate between the stimuli during pre-exposure, and had access to an unrestricted amount of the solution, they should display a preference for the sweet solution relative to the salty one. To the best of our knowledge, pre-exposure schedule effects on stimulus discrimination have not yet been tested in this simple and more ecological way.

### Method

#### Subjects and Apparatus

Subjects were 64 experimentally naïve male Wistar rats with a mean weight of 350 g (range 346–355 g) at the beginning of the experiment. Rats were individually housed in cages with food and water *ad libitum* in a room with constant temperature (24°C) and humidity (50%). The room was artificially illuminated under a 12 h-dark/light cycle with the light period beginning at 8:00 a.m. All experimental sessions were conducted with the animals in their home cages.

The stimuli used were two compound solutions, AX and BX, consisting of diluted plain water presented at room temperature through 50-ml plastic tubes, fitted with a metal spout. Stimulus X was always a solution containing hydrochloric acid (HCl) at 1%, whilst Stimulus A and B were (counterbalanced) a solution of 1% salt and 10% sugar, respectively, for half the rats in each group, and the reverse for the remaining half. The unconditioned stimulus (US) was an intraperitoneal injection of 0.15 M lithium chloride (LiCl) administered at 10 ml/Kg of body weight.

#### Procedure

The Animal Welfare Ethics Committee of the Universidad del Pais Vasco (Experiments 1 and 2) and the Universidad Autónoma de Chile (Experiments 3 and 4) approved the procedures employed in the current experiments. The rats were randomly assigned to eight equal groups (*n* = 8): CNC-P, INT-P, BLK-P, CTRL-P, CNC-U, INT-U, BLK-U, and CTRL-U.

##### Pre-exposure

All the rats, except those belonging to Groups CTRL-P and CTRL-U, received four AX and four BX presentations during pre-exposure. This phase lasted for 4 days, with the stimuli being presented during two daily 30-min drinking sessions (at 11:00 a.m. and 5:00 p.m.). These drinking sessions began by removing the bottles containing *ad libitum* plain water, and replacing these with two tubes, each containing 50 ml of flavored solution, one located on the right-hand side and the other on the left-hand side of the rats’ home-cages (30 cm apart). The sessions finished with the replacement of the two tubes with the bottle containing water *ad libitum*. For the CNC-P and CNC-U groups, in all the pre-exposure sessions one of the bottles contained the salty compound and the other the sweet compound. Half of the rats in these two groups first received the salty flavor on the right and the sweet flavor on the left (with the remaining rats receiving the reverse arrangement), the position of the solution being switched from trial to trial. For the INT-P and INT-U groups, the two tubes always contained the same solution, but it was switched from trial to trial. Half of the rats received the salty flavor on all of the morning sessions and the sweet flavor on the afternoon sessions, while for the remaining rats this arrangement was reversed. The BLK-P and BLK-U groups also received the same solution in both tubes. However, in these groups, half of the rats received the salty flavor on the first 2 days (on both the morning and afternoon sessions) and the sweet solution on the latter 2 days, while the other half received the reverse arrangement. Finally, the CTRL-P and CTRL-U groups always received plain water in both tubes. For half the rats in each group the salty solution was flavor AX and the sweet solution the flavor BX, while the reverse was true for the remaining rats. One hour after the last pre-exposure drinking session, the bottles containing *ad libitum* water were removed from the home cages. On the following 4 days, all the rats only had access to fluids during the 30 min morning and afternoon drinking sessions, in a single bottle placed in the location otherwise occupied by the standard water bottle.

##### Conditioning

The day after the last pre-exposure trial, all the rats received a single intraperitoneal injection of LiCl, half of them in the morning session and the other half in the afternoon session. For the CNC-P, INT-P, BLK-P, CTRL-P, and CNC-P groups, the injection was immediately preceded by the presentation of 10 ml of the AX solution in a single bottle, with the rats receiving 10 ml of plain water on the other session. Half of the rats from Groups CNC-U, INT-U, BLK-U, and CTRL-U received 10 ml of the AX solution in the morning, and 10 ml of plain water followed by the injection in the afternoon, whilst the remaining rats received 10 ml of water followed by the injection in the morning and 10 ml of the AX solution in the afternoon. The day following conditioning, all the rats received free access to plain water in the morning and afternoon drinking sessions.

##### Test

Over the next 2 days, all the rats had access to free consumption of the AX or BX solutions in the morning drinking session and water in the afternoon. Half of the rats from each group received the AX solution on the first day and the BX solution on the second, with the remaining rats receiving BX first and then AX. For all the subjects, the fluid was presented in a single bottle.

The amount of fluid consumed during the drinking sessions was calculated by weighing the drinking tubes before and after consumption and converting the difference to ml. An analysis of variance (ANOVA) was then conducted on the consumption values, adopting a statistical significance criterion of *p* < 0.05. Subsequent pair-wise comparisons between groups were conducted with the Tukey’s Honest Significant Difference (HSD) test.

### Results

**FIGURE 1:**
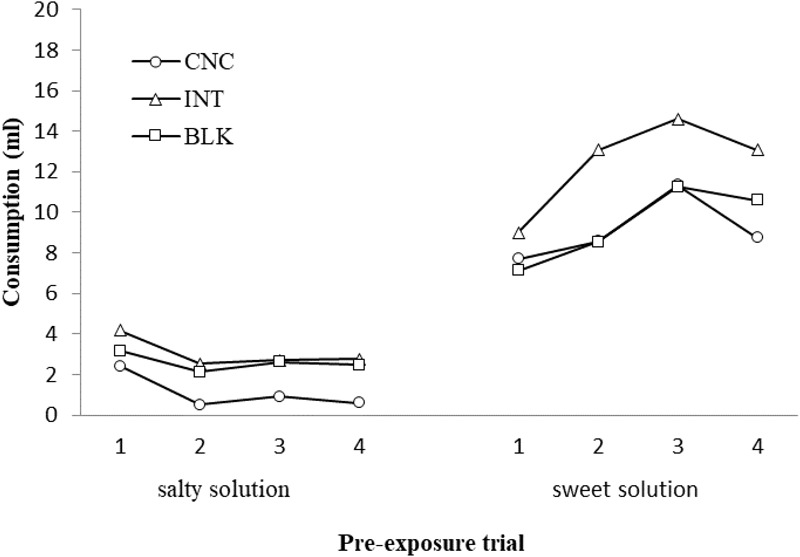
Consumption (ml) of the salty and sweet solutions for the concurrent, intermixed, and blocked pre-exposure conditions across blocks of pre-exposure trials in Experiment 1.

The mean consumption of the salty and sweet solutions during pre-exposure trials with the three schedules (Concurrent, Intermixed, and Blocked) is shown in **Figure 1**. Consumption of the sweet solution was always greater than consumption of the salty solution, with this consumption increasing in the former case and decreasing in the latter across the blocks of pre-exposure trials. Furthermore, consumption of the sweet solution was greater with the intermixed pre-exposure schedule than with the others, while consumption of the salty solution was lower with the concurrent schedule than the other schedules.

A 3 × 2 × 4 ANOVA with Schedule, Solution, and Trial as factors found a significant main effect of the three factors, Schedule, *F*(2,45) = 10.99, *p* < 0.001; Solution, *F*(1,45) = 4.15, *p* < 0.001; and Trial, *F*(3,135) = 4.89, *p* = 0.003. The Schedule × Solution, *F*(2,45) = 3.62, *p* = 0.035, and Solution × Trial, *F*(3,135) = 13.29, *p* < 0.001, interactions were also significant. A subsequent analysis of simple effects for these interactions confirmed the observations described above. Greater consumption of the sweet solution than the salty solution was found with all three pre-exposure schedules, *F*s(1,15) ≥ 95.89, *p*s ≤ 0.001, with pre-exposure conditions differing in both cases, *F*s(2,45) = 10.25, *p* < 0.001. Consumption of the sweet solution was greater with the intermixed than with the concurrent, *p* = 0.003, or the blocked schedule, *p* = 0.007, while consumption of the salty solution was lower with the concurrent than with the intermixed, *p* < 0.001, or blocked, *p* = 0.005, schedule. Consumption of the sweet solution was greater than the salty solution in all four blocks of pre-exposure trials, *F*s(1,47) ≥ 44.47, *p* ≤ 0.001, with the former increasing across blocks of trials, *F*(3,141) = 11.36, *p* < 0.010, while the latter decreased, *F*(3,141) = 3.95, *p* = 0.010.

On the conditioning trial, consumption of AX was 8.04 (SEM ± 1.36), 9.66 (SEM ± 1.29), 9.86 (SEM ± 1.53), and 8.77 (SEM ± 1.43) for the Concurrent, Intermixed, Blocked, and Control conditions, respectively. An ANOVA conducted with these consumption data found no significant effect of Schedule, *F*(3,60) = 0.35, *p* = 0.783.

**FIGURE 2:**
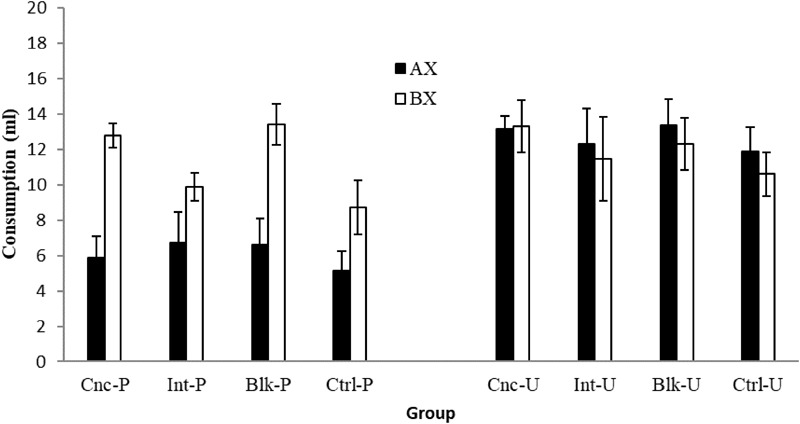
Consumption (mean ± SEM) of the AX and BX solutions during the test trials for the 8 groups of Experiment 1.

The mean consumption of AX and BX for the paired and unpaired groups during testing can be seen in **Figure 2**. Irrespective of the pre-exposure schedule, unpaired groups consumed a similar amount of AX and BX, but consumption of AX was always lower than consumption of BX for the paired groups. In this latter case, consumption of AX was similar in all groups, while consumption of BX appeared to be greater for the CNC-P and BLK-P groups than for the INT-P and CTRL-P groups. Furthermore, consumption of AX was, in general, lower for the paired than for the unpaired groups. However, consumption of BX was observed to be somewhat lower for the INT-P and CTRL-P groups than for the INT-U and CTRL-U groups, respectively, and very similar for the CNC-P and CNC-U, and BLK-P and BLK-U groups. A 4 × 2 × 2 ANOVA conducted with Schedule, Stimulus, and Conditioning as factors supported all the above observations except for the latter. The main effect of Conditioning, *F*(1,56) = 21.52, *p* < 0.002, and Stimulus, *F*(1,56) = 11.28, *p* = 0.001, as well as the interaction Stimulus × Conditioning, *F*(1,56) = 21.211, *p* < 0.001, were significant (remaining *F*s ≤ 0.89). A subsequent analysis of simple effects for the Stimulus × Conditioning interaction found lower consumption of AX than BX in the paired groups, *F(*1,31) = 41.28, *p* < 0.001, but not in the unpaired groups, *F*(1,31) = 0.616, *p* = 0.439. Furthermore, consumption of AX was lower in the paired than in the unpaired groups, *F*(1,62) = 45.57, *p* < 0.001, while consumption of BX did not differ reliably in the paired and unpaired groups, *F*(1,62) = 0.470, *p* = 0.496. Previous studies assessing the effect of the concurrent schedule on generalization have inferred its magnitude only from the consumption of BX (e.g., Alonso and Hall, 1999; Rodríguez and Alonso, 2008). Thus, although the Schedule × Stimulus interaction failed to reach significance, some planned comparisons were conducted in order to explore the differences in consumption observed for the paired groups. These analyses indicated that while the paired groups did not differ in their consumption of AX, *F*(3,28) = 0.26, *p* = 0.84, they differed in their consumption of BX, *F*(3,28) = 4.24, *p* = 0.014. In particular, consumption of BX was greater in Group BLK-P than in Group CTRL-P (*p* = 0.026).

### Discussion

In Experiment 1, rats consumed a greater amount of the sweet-acid solution than the salty-acid solution from the first pre-exposure trial. This initial discriminative response to the solutions seems to indicate that the rats were able to differentiate between them *a priori*, the sweet solution being more palatable than the salty solution. The consumption of the salty solution decreased in general during pre-exposure while consumption of the sweet solution increased. Thus, at least when rats were not water-deprived during pre-exposure, consumption of the less palatable solution might decrease unconditionally, while consumption of the more palatable solution increases. Experiment 1 also found a clear effect of the schedule on the pattern of consumption elicited by the salty and sweet solutions during pre-exposure. Rats consumed less of the salty solution during concurrent than intermixed or blocked pre-exposure. However, the sweet solution was more readily consumed during intermixed than concurrent or blocked pre-exposure. If we accept that the stimuli were readily discriminated *a priori*, it is possible that the schedule affected consumption of the most and least palatable stimuli not by increasing stimulus differentiation, but rather through some other, as yet unspecified, effect of the schedule. This issue will be taken up again in the General Discussion.

On the test, all the rats from the paired groups drank less of the aversively conditioned flavor AX than the BX flavor. Again this discriminative response would be possible only if the stimuli were differentiated. Rats from the unpaired groups did not differ in their consumption of AX and BX. Given that no stimulus was conditioned in these groups, there would be no reasons to expect any difference in consumption of AX and BX even when they are discriminable (note that half of the rats that received the AX compounds were given the sweet solution and the other half received the salty solution). Consumption of AX was lower in the paired than in the unpaired groups, indicating that an aversion to AX was successfully established in these cases. And importantly, there were no significant differences between the paired and unpaired groups in terms of their consumption of BX. Since consumption of BX in the unpaired groups would be free from any generalization of conditioned aversion, this last result may indicate that the paired groups did not generalize the conditioned aversion from AX to the other stimulus, BX. In brief, none of the results obtained on the test appear to indicate generalization between AX and BX in the paired groups, and we can thus rule out the possibility that generalization was differentially affected by the pre-exposure schedules. Nevertheless, in the paired groups, consumption of BX was observed to be greater after blocked pre-exposure to the stimuli relative to the control group that did not receive prior pre-exposure to the stimuli. Taking the consumption of BX alone, it might be possible to conclude that the blocked but not the intermixed and concurrent pre-exposure schedules reduced generalization between the stimuli (relative to the control group not receiving pre-exposure to the stimuli). Such a conclusion would be incompatible with the results produced by previous studies (Alonso and Hall, 1999; Rodríguez and Alonso, 2008; Rodríguez et al., 2008), in which rats drank less BX after concurrent than intermixed or blocked pre-exposure, concluding that generalization was greater in the former than in the latter cases. However, the results of Experiment 1 appear to suggest that consumption of BX might not be a net indicator of generalization between the stimuli. Given that there is no sign of generalization between the stimuli, and taking into account that pre-exposure schedules might directly affect consumption, it seems likely that differences in consumption of BX between the groups could reflect some effect of the schedules on consumption rather than on stimulus generalization.

Presumably, the discrepancy between our findings and those of previous studies regarding the consumption of BX might be related to the state of fluid deprivation during pre-exposure. However, it should be noted that this and the previous studies also differed in terms of the amount of solution that was made available during pre-exposure. In the experiments conducted previously, rats received only a small and fixed amount of the solutions during pre-exposure (10 ml), and these were usually fully consumed given that the rats were water deprived (but see Alonso and Hall, 1999, Experiment 1a). But in Experiment 1 of the present study, rats had free access to the flavored solutions. Therefore, the main aim of Experiment 2 was to test whether the principal findings of Experiment 1 could be replicated if rats received a fixed amount of the solutions during pre-exposure, as in previous experiments reported in the literature.

## Experiment 2

The methods used in Experiment 1 and those employed in previous studies (e.g., Rodríguez and Alonso, 2008; Rodríguez et al., 2008, but see Alonso and Hall, 1999), differed in two important ways. Firstly, in Experiment 1 (but not in previous studies), the rats were not in a state of water deprivation during pre-exposure (but see Alonso and Hall, 1999). And secondly, in Experiment 1 (but not in the previous studies), rats were allowed free access to the solutions during pre-exposure instead of a limited, fixed amount of the flavor. In Experiment 2, rats in the paired groups also received a limited amount of the solution during pre-exposure in order to see if such a variable could play a role in our failure to observe the expected effect of enhanced generalization after concurrent pre-exposure.

### Method

#### Subjects and Apparatus

Subjects were 32 experimentally naïve male Wistar rats with a mean *ad libitum* weight of 398 g (range 391–405 g) at the beginning of the experiment. The stimuli, apparatus, and all other details not specified here were identical to those described for Experiment 1.

#### Procedure

Rats were randomly assigned to four equal groups (*n* = 8), CNC-P, INT-P, BLK-P, and CTRL-P, which, as in Experiment 1, differed only in terms of the pre-exposure schedule received (Concurrent, Intermixed, Blocked, or No exposure, respectively). However, unlike in the previous experiment, in this case the rats received only 10 ml of solution on each pre-exposure trial (5 ml in each bottle). All other procedural details not specified here were identical to those described for Experiment 1.

### Results

**FIGURE 3:**
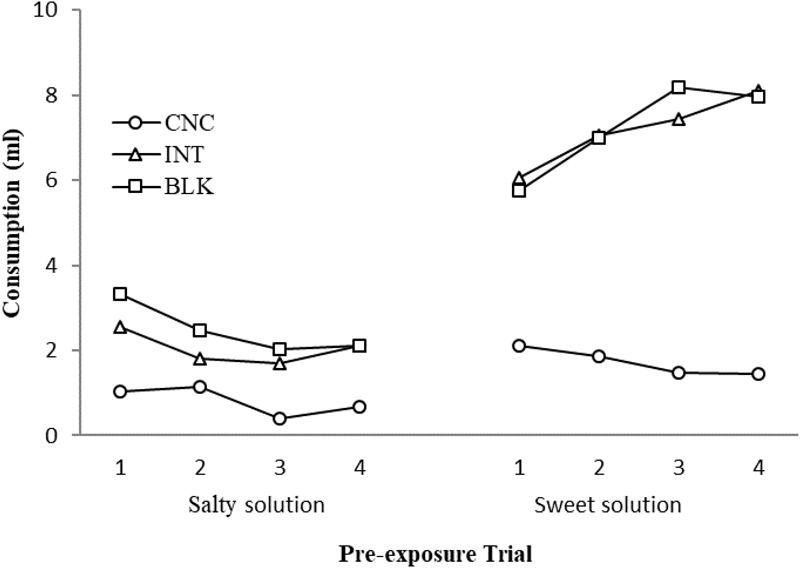
Consumption (ml) of the salty and sweet solutions for the concurrent, intermixed, and blocked pre-exposure conditions across blocks of pre-exposure trials in Experiment 2.

Consumption of the salty and sweet solutions during pre-exposure with the Concurrent, Intermixed, and Blocked schedules can be seen in **Figure 3**. Again, consumption of the salty solution was lower than the sweet solution and in this case, consumption of both solutions was observed to be lower with the concurrent than with the intermixed or blocked schedules. In general, consumption of the salty solution also decreased somewhat across blocks of trials (with all schedules), while consumption of the sweet solution was observed to increase in general (with all schedules). A 3 × 2 × 4 ANOVA conducted with Schedule, Solution, and Trial as the factors found significant main effects of Schedule, *F*(2,21) = 47.66, *p* < 0.001, and Solution, *F*(1,21) = 262.440, *p* < 0.001. Of the interactions, Schedule × Solution was found to be significant, *F*(2,21) = 36.94, *p* < 0.001, remaining, *F*s ≤ 2.70. Subsequent analyses of simple effects for the interaction found that for all schedules, consumption of the sweet solution was always greater than consumption of the salty solution, *F*s(1, 7) ≥ 22.50, *p*s ≤ 0.002. Groups differed in their consumption of both the salty, *F*(2,21) = 7.39, *p* = 0.004, and the sweet solution, *F*(2,21) = 70. 77, *p* < 0.001, with consumption always being lower for subjects that received the concurrent schedule compared with the intermixed or blocked schedules, *p*s ≤ 0.032.

On the conditioning trial, consumption of AX was 6.37 (SEM ± 1.39), 5.48 (SEM ± 1.15), 5.70 (SEM ± 1.32), and 5.57 (SEM ± 1.0) for the CNC-P, INT-P, BLK-P, and CTRL-P groups, respectively. An ANOVA conducted on these consumption scores revealed that differences between the groups were not significant, *F*(3,28) = 1.61, *p* = 0.208.

**FIGURE 4:**
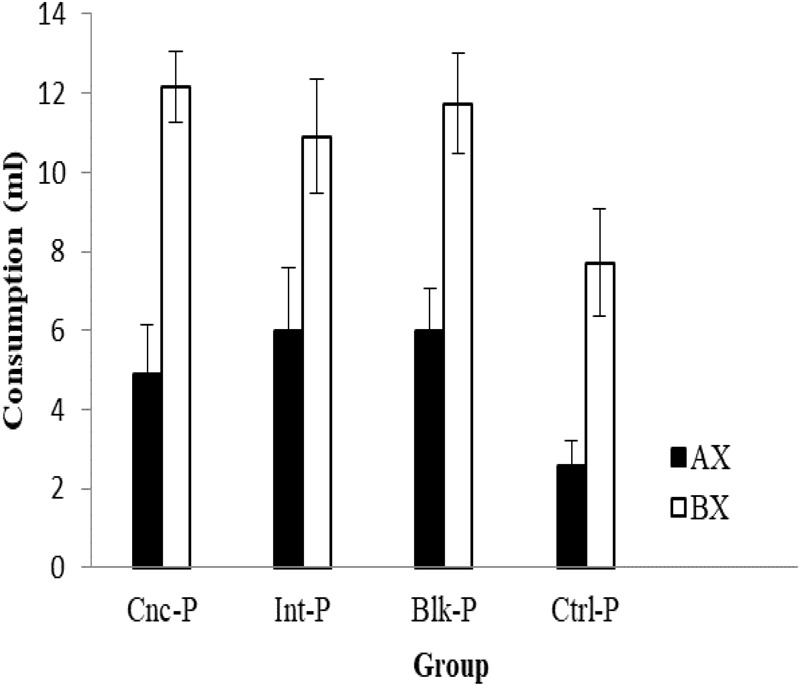
Consumption (ml, mean ± SEM) of the AX and BX solutions during the test trials for the four groups of Experiment 2.

Finally, consumption of AX and BX on test for all four groups can be seen in **Figure 4**. In all four groups, consumption of AX was lower than consumption of BX, and in this case, consumption of both AX and BX was observed to be lower in Group CTRL than in the others. A 4 × 2 ANOVA conducted with Schedule and Stimulus as factors found only the main effect of Stimulus to be significant, *F(*1,28) = 81.46, *p* < 0.001, although the effect of Schedule also approached significance, *F*(3,28) = 2.81, *p* = 0.057. The interaction Schedule × Stimuli was far from reliable, *F*(3,28) = 0.687, *p* = 0.567 and, if anything, a subsequent pair-wise comparison between groups revealed that only the CTRL and BLK groups differed marginally (*p* = 0.077) in terms of their general consumption, as in Experiment 1.

### Discussion

As observed in Experiment 1, consumption of the sweet solution was greater than consumption of the salty solution from the outset of pre-exposure, with consumption of the salty solution being lower during concurrent than intermixed or blocked pre-exposure. In this case, consumption of the sweet solution was also lower during concurrent than intermixed or blocked pre-exposure. This result, however, might readily be explained in terms of a procedural artifact. In order to provide the same overall amount of flavored solution during pre-exposure for all the groups, rats given concurrent pre-exposure to the stimuli received only 5 ml of each solution on each trial while rats receiving intermixed or blocked pre-exposure received 10 ml (5 ml in each tube). Thus, the rats receiving the concurrent schedule were never able to drink more than 5 ml of the sweet solution during pre-exposure. The consumption of the sweet solution in the groups receiving the intermixed and blocked schedules was similar and asymptotic (note that the last ml of fluid in the tube is usually inaccessible). It thus seems likely that the greater consumption of the sweet solution observed in the intermixed group relative to the other groups in Experiment 1 was not observed here because an insufficient amount of solution was available to reveal this difference.

It should perhaps be noted that, regarding the ability to discriminate the stimuli, lower consumption of the stimuli during concurrent pre-exposure relative to the intermixed or blocked schedules would lead, if anything, to poorer stimulus discrimination in the former than in the latter cases. In spite of this, however, on the subsequent test, nothing in our results indicated poorer discrimination or greater generalization between the stimuli after concurrent exposure in comparison with intermixed or blocked pre-exposure. All rats consumed less of the conditioned stimulus (CS) flavor (AX) than BX, indicating good discrimination between the stimuli in all cases. According to some theoretical analyses, generalization between the stimuli should be increased by the concurrent schedule because this arrangement should boost the establishment of excitatory links between the stimuli and strengthen conditioning of their common elements. If this were the case, consumption of BX should have been lower after concurrent than intermixed or blocked pre-exposure. But in the present experiment, consumption of both AX and BX was similar after concurrent and intermixed or blocked pre-exposures. Again, this experiment also provides no evidence that stimulus generalization differs according to the pre-exposure schedule and, if anything, the blocked schedule may have increased the general consumption of the solutions relative to the control group. It might be worth noting, however, that contrary to our expectations, consumption of BX appeared to be somewhat higher following concurrent exposure compared with the intermixed schedule, although this difference was not significant (see also Experiment 1).

In brief, the results of the two experiments presented above appear to be incompatible with the idea that concurrent pre-exposure to the stimuli increases generalization between them, or at least, not in all cases. Such a result would be largely unexpected, given that two previous studies (Rodríguez and Alonso, 2008; Rodríguez et al., 2008) found greater generalization of a conditioned taste aversion after concurrent exposure in comparison with intermixed pre-exposure. Further, according to some authors, associative mechanisms would be operating to increase generalization after concurrent pre-exposure. In particular, the latter arrangement should facilitate the establishment of excitatory links between the pre-exposed stimuli. Furthermore, according to previous evidence, the common elements of the stimuli might have acquired a stronger aversion during conditioning following concurrent pre-exposure due to an attenuation of latent inhibition (i.e., Bennett and Mackintosh, 1999; Rodríguez and Alonso, 2008). Given that the main procedural difference between those experiments reporting increased generalization after concurrent pre-exposure and those reported here was the state of fluid deprivation, it is reasonable to suppose that this factor could be responsible for the discrepancy between the results of these studies. To be more specific, it is well-established that contextual changes after pre-exposure usually attenuate latent inhibition (i.e., Hall and Honey, 1989) and, it would be expected that the change in the state of deprivation between the pre-exposure and conditioning phases in our experiments might be able to change the internal context of the animals. If this were the case, such a contextual change might serve to attenuate latent inhibition of AX for subjects in all the pre-exposure conditions. It might then be possible to argue that the differences in latent inhibition expected for the common elements of the stimuli according to the pre-exposure schedule might also be attenuated, thus reducing the likelihood of detecting subsequent differences in the generalization of the conditioned taste aversion. This possibility is supported by the fact that in the previous experiments the subjects who received pre-exposure to the stimuli did not differ from the non-preexposed control group in terms of test consumption of AX, as would be expected on the basis of a very robust effect such as latent inhibition. Following this logic, the aim of Experiment 3 was to explore the possible role played by the deprivation state of the animals in observing an effect of intermixed and concurrent pre-exposure on the generalization of a taste aversion. In order to meet the ethical requirement of reducing the number of animals used, in the following experiment only were maintained such pre-exposure conditions critical for the theoretical discussion, that is, those subjects in the intermixed and concurrent pre-exposure conditions.

## Experiment 3

The chief aim of Experiment 3 was to test the effect of water deprivation during intermixed and concurrent pre-exposure on the subsequent generalization of a conditioned taste aversion, and in particular whether or not the enhanced generalization observed after concurrent pre-exposure might occur only in deprived rats. This latter result would be expected if such an increase in generalization were due to an attenuation of latent inhibition, where the change in the deprivation state might disrupt latent inhibition for both pre-exposure conditions. In order to test this possibility, a conditioned taste aversion was established to the compound AX, as in the previous experiments, followed by a consumption test in which animals are presented with this flavor and a second, similar flavor, BX. In a previous phase, the rats received either concurrent (CNC conditions) or intermixed (INT conditions) pre-exposure to the stimuli, and were either water deprived or not (D and ND conditions respectively) during pre-exposure.

### Method

#### Subjects and Apparatus

Subjects were 32 naïve male Sprague Dawley rats with a mean *ad libitum* weight of 299 g (range 286–312 g) at the beginning of the experiment. The stimuli, apparatus, and other details not specified were identical to those described for Experiment 2.

#### Procedure

The rats were randomly assigned to four equal groups (*n* = 8): CNC-D, INT-D, CNC-ND, and INT-ND. For the deprived conditions, 4 days before the pre-exposure phase, access to water was limited to two daily 30 min sessions. This drinking schedule was also maintained during pre-exposure. However, for the non-deprived rats, fluid was always available until the conditioning phase. In this case, during the pre-exposure phase the tubes containing the flavored compounds replaced the bottles containing plain water, with the latter being returned after pre-exposure. Given that the aim of this experiment was to specifically test the effect of deprivation, the level of fluid available in each trial for both the intermixed and concurrent pre-exposure conditions was 10 ml (5 ml in each tube), being the fluid the same for the intermixed conditions and different for the concurrent conditions.

### Results

The consumption of the salty and sweet solutions for the four groups can be seen in the upper part of **Table 1**. In general, and as expected, the consumption of both solutions was greater for those subjects in the deprived conditions than in the non-deprived conditions. Furthermore, for the non-deprived conditions the consumption of the sweet solution appeared to be greater than the salty solution, as observed in previous experiments; however, for the deprived conditions this difference was less apparent. Furthermore, as found in Experiment 1, in the non-deprived conditions the consumption of the sweet solution appeared to increase with the intermixed schedule whilst consumption of the salty solution seemed to decrease with the concurrent schedule. A 2 × 2 × 2 × 4 ANOVA with Deprivation (Deprived vs. Non-Deprived), Schedule, Flavor, and Trial as the factors found significant main effects of Deprivation, *F*(1,28) = 148.26, *p* ≤ 0.001, and Flavor, *F*(1,28) = 22,87, *p* ≤ 0.001. Furthermore, the interactions Deprivation × Schedule, *F*(1,28) = 4.67, *p* = 0.039, Flavor × Deprivation, *F*(1,28) = 8.23, *p* = 0.008, Deprivation × Trial, *F*(3,84) = 6.91, *p* ≤ 0.001, and Schedule × Trial, *F*(3,84) = 19.29, *p* ≤ 0.001, were also significant (remaining, *F*s ≤ 1.95). The subsequent analysis of simple effects found that rats in the deprived conditions consumed more than those in the non-deprived conditions, both with the Concurrent, *F*(1,15) = 36.78, *p* ≤ 0.001, and Intermixed *F*(1,15) = 161,44, *p* ≤ 0.001, schedules, but the effect of schedule was significant only for the deprived rats, *F*(1,15) = 9.38, *p* = 0.008. Furthermore, rats in the deprived conditions drank more of both the salty, *F*(1,31) = 169.61, *p* ≤ 0.001, and sweet solutions, *F*(1,31) = 32.82, *p* ≤ 0.001. However, the consumption of the sweet solution was greater than the salty solution only for the non-deprived rats, *F*(1,15) = 40.58, *p* ≤ 0.001. In addition, the effect of Deprivation was significant on the four pre-exposure trials, *F*s ≥ 15.24, with the effect of trial only being significant for the deprived rats, *F*(3,45) = 3.24, *p* = 0.031. Finally, the general effect of Schedule was significant for the first, *F*(1,31) = 4.46, *p* = 0.043, and latter pre-exposure trials, *F*(1,31) = 21.145, *p* ≤ 0.001, with a significant effect of trial for the Deprived, *F*(3,45) = 3.24, *p* = 0.031, but not for the Non-Deprived rats, *F*(3,45) = 2.10, *p* = 0.112.

**Table 1:** Consumption (ml) of the salty and sweet solutions during the pre-exposure trials for all the groups of Experiments 3 and 4.

Group	Salty 1	Salty 2	Salty 3	Salty 4	Sweet 1	Sweet 2	Sweet 3	Sweet 4
Experiment 3					
CNC-D	10.00	7.84	9.42	4.47	9.79	6.34	10.00	7.64
INT-D	7.75	8.53	9.63	9.82	9.14	9.99	9.99	10.00
CNC-ND	3.10	2.24	2.92	2.05	5.00	5.00	5.01	5.00
INT-ND	1.36	1.62	1.18	4.35	2.61	4.97	4.81	8.42
Experiment 4					
CNC-1B	4.49	4.69	5.00	5.00	4.32	4.72	5.00	4.90
CONC-2B	4.44	5.00	5.00	5.00	4.26	4.89	5.00	4.77
INT-1B	8.27	10.00	10.00	10.00	8.06	8.61	9.83	9.68
INT-2B	7.18	10.00	10.00	9.64	7.59	9.71	9.42	9.80

On the conditioning trial, consumption of AX was 8.88 (SEM ± 0.24), 8.67 (SEM ± 0.194), 9.34 (SEM ± 0.18), and 8.86 (SEM ± 0.17) for the CNC-D, CNC-ND, INT-D, and INT-ND groups, respectively. An ANOVA conducted on these consumption scores revealed no significant differences between the groups, *F*(3,28) = 1.97, *p* = 0.141.

**FIGURE 5:**
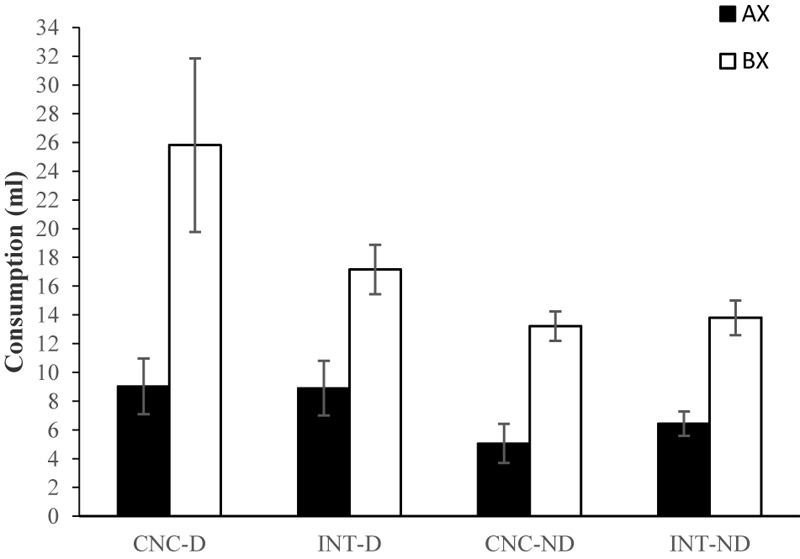
Consumption (ml, mean ± SEM) of the AX and BX solutions during the test trials for the four groups of Experiment 3.

**Figure 5** shows the consumption of the compound AX and BX on the test for the four experimental conditions. In general, consumption of both AX and BX was greater for rats in the deprived conditions compared with the non-deprived conditions, with consumption of BX being generally higher than that of AX. Consumption of BX appeared to be particularly high for the CNC-D groups in comparison with the other groups.

A 2 × 2 × 2 ANOVA conducted on these data with Deprivation (Deprived vs. Non-Deprived), Schedule, and Stimulus found significant main effects of Deprivation, *F*(1,28) = 10.46, *p* = 0.003, and Stimulus, *F*(1,28) = 29.34, *p* < 0.001 (remaining, *F*s ≤ 2.38). Planned comparisons to examine possible differences in the consumption of AX and BX according to the schedule found an effect of the schedule for the consumption of BX, *F*(3,28) = 3.20, *p* = 0.038, but not for AX, *F*(3,28) = 1.52, *p* = 0.229, with consumption of BX being higher for subjects given the concurrent schedule than those given the intermixed schedule.

### Discussion

As observed in Experiments 1 and 2, Experiment 3 found that when rats were not deprived during pre-exposure, they displayed a preference for the sweet solution over the salty solution during pre-exposure, which clearly indicates that they can discriminate the compounds *a priori*. The three experiments appeared to be consistent in this regard. Unsurprisingly, deprived rats tend to consume all the fluid available and thus the preference would not be detected when a deprivation regime is introduced in the taste aversion preparation. The fact that the deprived rats drank more than the non-deprived rats during pre-exposure requires no special comment and, since the opportunity for consumption in this experiment was also limited, the present experiment presented no opportunity to replicate the principal findings of Experiment 1. Thus, no further comments regarding the pre-exposure phase are necessary.

In addition, Experiment 3 found a general effect of deprivation on the test that was reflected in the greater consumption of both AX and BX for the deprived rats. Although all the rats were deprived from the conditioning phase, the most parsimonious explanation for this finding was that even in this case the general state of deprivation was greater for the rats that were maintained in a state of deprivation for a longer time period. Notwithstanding, maybe one might also consider the possibility that the incentive value of the flavors might to differ according to the previous status of deprivation, being the hedonic impact higher for the deprived than non-deprived rats. If this was the case, one might also to expect a greater consumption for the deprived than non-deprived rats both during conditioning and test. But because during conditioning only a limited amount of fluid was available, the effect would have been observed only during the test. In any case, these findings are also entirely consistent with the notion that the change in the internal context for the non-deprived rats might attenuate the latent inhibition suffered by AX, increasing the conditioning of this compound and hence the generalization of the aversion to BX. Given that this experiment does not allow us to distinguish between these alternatives, on the basis of parsimony it seems reasonable to accept the first explanation. But whatever the explanation, the results of this experiment were largely unexpected. In general, but particularly for the deprived rats, the consumption of BX was higher for those rats in the concurrent pre-exposure condition than those given the intermixed schedule. This result is precisely the opposite of that expected according to the literature and the associative mechanisms described previously. Thus, how might this result be interpreted?

Before entering into further theoretical speculation, a final experiment was conducted in order to examine the effect of the concurrent and intermixed pre-exposure schedule effects in deprived rats with a conditioned taste aversion preparation more similar to that employed by Rodríguez et al. (2008). The rationale for this is that first, in the previous experiments we only employed a single conditioning trial while Rodriguez et al. used two. If the effect of the concurrent schedule reported by these authors relies on the attenuation of latent inhibition of the common elements produced by this schedule, then it is possible that the reduction in the general level of conditioning hindered the possibility of finding the effect. Secondly — and also related to the potential attenuation of latent inhibition for the common elements with the concurrent schedule — another procedural detail that might serve to obscure the effect is the change in the number of bottles presented during the pre-exposure and conditioning phases. In our experiments, but not in those reported previously, during conditioning AX was presented only in one tube while during pre-exposure the compounds were always presented in two tubes. Thus, a change in context might not only be provided by the deprivation state, but also by a change in the number of bottles presented between pre-exposure and conditioning; whilst the former represents a change in the internal context of the animal, and the latter an external change, both of these modifications could potentially disrupt latent inhibition as suggested previously.

## Experiment 4

Experiment 4 aimed to test whether or not the enhanced generalization expected after concurrent pre-exposure relative to the intermixed schedule might be observed in a preparation more similar to that employed by Rodriguez et al. In particular, we wanted to verify whether a contextual change such as the one that might be provided by changing the number of bottles between pre-exposure and conditioning could mask the increment in generalization expected for the concurrent schedule, and thus supply an explanation for our apparent failure to find this effect.

Experiment 4 therefore included two conditions for which the compound AX was presented in two bottles during conditioning, in the same way as during pre-exposure. The other two groups of the experiment received identical treatment to Groups CNC-ND and INT-ND in the previous experiments except that in this case, all rats received two conditioning trials and the amount of fluid available in each tube was 5 ml. Whilst it is true that changing two factors from Experiment 3 makes it difficult to identify which of these is important, it should be noted that the general aim was to more closely follow the procedure that has been shown to generate the concurrent/intermixed effect described previously.

### Method

#### Subjects and Apparatus

Subjects were 32 naïve male Sprague Dawley rats with a mean *ad libitum* weight of 284 g (range: 222–331 g) at the beginning of the experiment. Any other details of the apparatus, stimuli, and procedures not specified here were identical to those of Experiment 2.

#### Procedure

Rats were randomly assigned to four equal groups (*n* = 8): CNC-1B, INT-1B, CNC-2B, and INT-2B. During pre-exposure, all the rats received 5 ml of the compounds in each bottle, these being the same for the intermixed condition and different for the concurrent. For half the participants in the intermixed and concurrent pre-exposure conditions the compound AX was presented in one bottle for conditioning, as in the previous experiments (Conditions-1B) while for the remaining half the compound AX was presented in two bottles (as in the experiments reported in other studies). Furthermore, in this experiment all the rats received two conditioning trials rather than one. All the rats then received a consumption test with the compound AX followed by a single generalization test with the BX flavor.

### Results

The consumption of both the salty and sweet solutions during pre-exposure is displayed in **Table 1**. Given the fact that the available amount of each flavor for the subjects in the INT condition was twice that given to those in the concurrent condition, the overall amount consumed was greater in the former than in the latter case. The consumption of the sweet solution appeared to be somewhat higher than the salty solution but in general, from the second trial, the rats consumed most of the fluid available. A 2 × 2 × 4 ANOVA conducted on these data with Schedule, Solution, and Trial as the factors found a significant main effect of Schedule, *F*(1,30) = 1.975, 67, *p* ≤ 0.001, Solution, *F*(1,30) = 9.17, *p* ≤ 0.005, and Trial, *F*(3,30) = 44.67, *p* ≤ 0.001. There was also a significant interaction between Schedule and Trial, *F*(3,30) = 12.92, *p* ≤ 0.001 (remaining, *F*s ≤ 1.61). The subsequent analysis of this interaction revealed a significant effect of Schedule for the four pre-exposure trials, *F*s ≥ 152.710, and an effect of Trial for both Schedules, *F*s ≥ 27.34.

**FIGURE 6:**
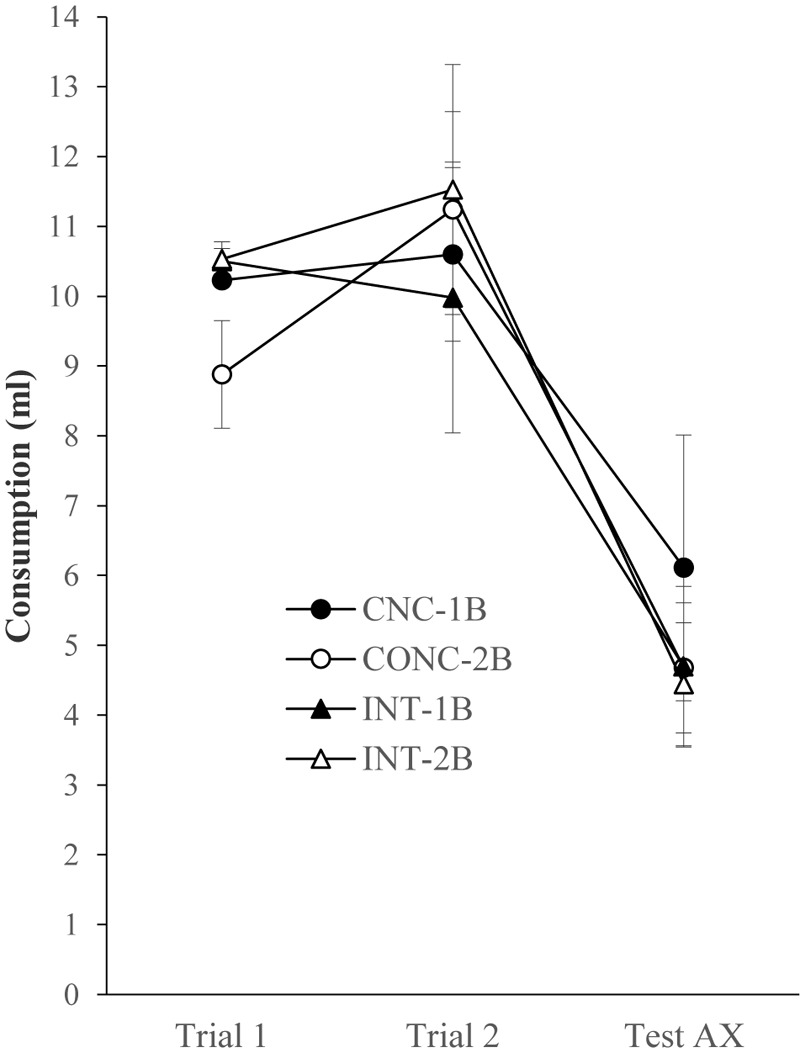
Consumption (ml, mean ± SEM) of the AX solution during the two conditioning trials and the test for the four groups of Experiment 4.

**Figure 6** shows the mean consumption of compound AX on the two conditioning trials and the test for the four experimental conditions. It appears that consumption of AX was similar for the four conditions, and it declined across the trials in all cases. A 2 × 2 × 3 ANOVA conducted on these data with Schedule, Number of bottles, and Trial as the factors found a significant main effect of Trial, *F*(1,28) = 41.249, *p* ≤ 0.001 (Remaining *F*s ≤ 1.16).

**FIGURE 7:**
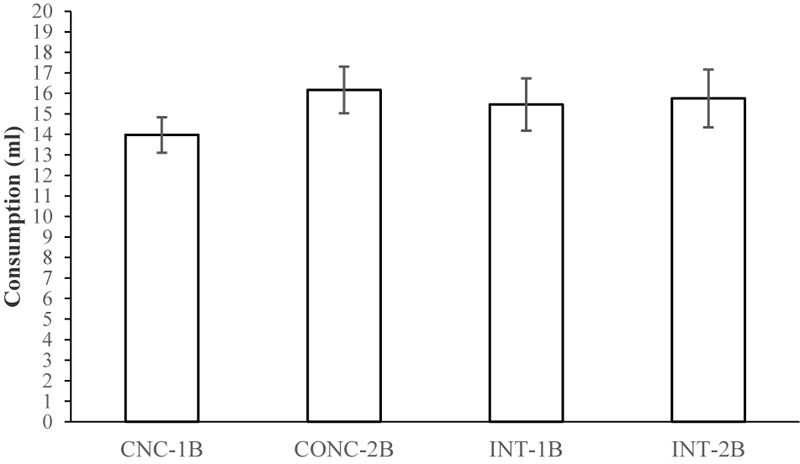
Consumption (ml, mean ± SEM) of the BX solution during the test for the four groups of Experiment 4.

Finally, **Figure 7** shows the mean consumption of the BX compound for the four experimental groups. It is clear that consumption of this compound was similar for all groups. A 2 × 2 ANOVA with Schedule and Bottle as the factors failed to find any significant main effects of these factors or any interaction between them, *F*s ≤ 1.11.

### Discussion

The final experiment failed to find an effect of either the schedule or the change in the number of bottles presented during the pre-exposure and conditioning phases on conditioning or generalization. Thus, it might be thought that this supposed contextual change was either ineffective or was of insufficient strength to be detected with our experimental procedure.

Again, this experiment also found no evidence of an enhancement in generalization after concurrent pre-exposure compared with the intermixed schedule. It is possible that our failure to find an effect that has previously been reported in the literature could still lie in parametric factors not considered in the present study. However, if the incremental effect of concurrent pre-exposure on generalization can only be obtained under a very specific set of conditions such as those reported in previous experiments, then perhaps the generality of the effect should be questioned. This is particularly important when we consider that it is precisely in this efect where we find a mismatch between the results from human and non-human animal experiments, and that there are only very few studies addressing this issue using animal experiments of the sort carried out in the present paper.

## General Discussion

The present study was conducted with the principal aim of examining whether the state of water deprivation exerts an effect on stimulus generalization in rats and whether such an effect might modulate the effects of pre-exposure schedules that have been established in the literature. Recently, several studies have reported important interactions between the pre-exposure schedules and the instructions given to people during pre-exposure. Thus, we may question whether these general effects of pre-exposure can be regarded as unequivocal. If this is true for humans, then perhaps other factors might be modulating the pre-exposure effects for other animals with other experimental paradigms, and these factors could be responsible certain discrepancies between the results of studies conducted with human and non-human animals. The most controversial findings in this regard are those related to the concurrent pre-exposure schedule, and therefore the present series of experiments set out to specifically examine the effect of this pre-exposure schedule on stimulus generalization.

Before entering into a discussion of the results that are relevant to the principal aims of this study, it is worth mentioning some rather unexpected but very interesting findings regarding the effects of the pre-exposure schedule on consumption in thirsty rats. Experiments 1 and 2 found greater consumption of the sweet-acid solution than the salty-acid solution from the first pre-exposure trial. This result indicates that both solutions were initially differentiated, given that the sweet solution is more palatable than the salty solution. In Experiment 1, consumption of the salty solution was reduced in general during pre-exposure, while consumption of the sweet solution increased. Because the rats were not thirsty during pre-exposure, these results might easily be explained in terms of the palatability of the solutions. Rats would progressively avoid consuming the less palatable solution, while increasing their consumption of the more palatable flavor. But rather more difficult to explain is the observation that consumption of the least and most palatable solutions differentially changed according to the pre-exposure schedule. Experiments 1 and 2 found that consumption of the less palatable solution was lower with the concurrent schedule than the other schedules. This result might perhaps be regarded as an effect of sensory contrast, consumption of the more palatable solution serving to devalue the less palatable flavor. Sensory contrast would be hindered, however, during intermixed pre-exposure. Thus, the greater consumption of the sweet solution during intermixed than concurrent or blocked pre-exposure could not be explained in the same way. Given that the rats were able to differentiate the stimuli *a priori*, it seems unlikely that these pre-exposure schedule effects on consumption could arise from increments in stimulus differentiation. It is possible, however, that the different pre-exposure schedules might change the perception of the stimuli in a way that is distinct from increasing their discriminability. For example, it is possible that some perceptual learning mechanism that is modulated by the pre-exposure schedules could change the palatability of the stimulus. This suggestion, however, is merely speculative, and it seems reasonable to avoid any further development of this hypothesis until the appropriate experiments have been conducted to address the issue. The results for pre-exposure obtained in Experiments 3 and 4 were constrained by the limited amount of flavors available during pre-exposure and the state of water deprivation, as usually occurs in standard conditioned taste aversion procedures. These findings therefore are unable to shed further light on this issue. Nonetheless, the results from the first experiments might be regarded as a first step toward an interesting and more ecological way to assess the pre-exposure schedule effects in non-human animals, which might not be limited to effects on generalization.

Irrespective of the pre-exposure schedule received beforehand, and even when rats had no previous experience with the stimuli (CTRL groups, Experiments 1 and 2), test consumption of the conditioned flavor AX was lower than consumption of the other, similar, non-conditioned flavor BX. Thus, these latter results appear to confirm that rats were able to differentiate the stimuli even without pre-exposure. As acknowledged previously, generalization between the stimuli might be increased or reduced by different means and, in this regard, similar responses to different stimuli would not necessarily imply that the stimuli were not discriminable. However, a clear discriminative response to the stimuli on test, as found in the experiments reported here, would clearly only be possible if the stimuli had been discriminated. Furthermore, Experiment 1 found that the paired and unpaired groups did not differ in their consumption of BX. For the unpaired groups, consumption of BX would be free from generalization because AX was never conditioned. Thus it might be suggested that similar consumption of BX in the paired groups indicates that in these cases, there was no generalization of the aversion from AX to BX. This finding precludes the possibility that generalization between the stimuli would always increase after the concurrent schedule, as would be expected if excitatory links between the stimuli had been established, or their common elements had been strongly conditioned (e.g., Rodríguez and Alonso, 2008; Rodríguez et al., 2008).

Regarding the specific effect of the pre-exposure schedule on test, Experiment 1 found greater consumption of BX on the generalization test after concurrent or blocked pre-exposures to AX and BX, and in comparison with the control condition that had received no pre-exposure to any of the stimuli. If only this latter result were taken into consideration, one might conclude that concurrent and blocked pre-exposure (although perhaps not the intermixed schedule), may reduce generalization between the stimuli for no-thirsty rats. Nonetheless, the absence of any evidence of generalization between the stimuli (see Experiment 1) raises doubts about this conclusion. In the light of the findings obtained during pre-exposure, the results, on the whole, appear to indicate that the pre-exposure schedule also affected consumption of the solutions on test over and above their potential effects on generalization. Although only speculative at present, pre-exposure schedule effects could potentially serve to modulate stimulus palatability, or interact with conditioning, thus explaining such differences in the consumption on test. Again, further research is needed to directly address this possibility. Nonetheless, one of the merits of the present study is that it includes for the first time the unpaired control groups needed to address this hypothesis. Without these control groups, it is not possible to establish whether differences in consumption of the test stimulus might be related to generalization or some other factor that could potentially affect consumption of the test flavor.

Experiments 3 and 4 consistently failed to find any evidence for the expected increase in generalization after concurrent pre-exposure relative to intermixed exposure when the rats were deprived during pre-exposure, as is a routine feature of this type of experimental procedure. Experiment 3 showed a general effect of the deprivation state on increasing the consumption of the conditioned flavor, AX and the test flavor, BX. As discussed above, this finding might be interpreted in several ways. It is possible that for the non-deprived rats the possible change in the internal context that might be caused by the introduction of deprivation between the pre-exposure and conditioning phases might disrupt latent inhibition of AX. In this case, the aversion acquired by AX — and hence generalization to BX —would be stronger for rats in the non-deprived conditions. However, in the absence of independent evidence for this possibility, the more parsimonious explanation is that consumption was generally higher for the deprived rats because they were subjected to a limited drinking schedule for a longer period of time. Surprisingly, in Experiment 3 consumption of BX was higher after concurrent than intermixed pre-exposure for deprived rats. Given the absence of any differences in consumption of AX between the groups in the intermixed and concurrent pre-exposure conditions, this result might be taken to indicate that, for the deprived rats, given the similar levels of conditioning to AX, the generalization of the aversion to BX was less after concurrent pre-exposure. Clearly, this result is exactly the opposite of that reported by Rodríguez et al. (2008), and was largely unexpected, although it is perhaps worth noting that in our previous experiments such a tendency was also consistently observed. Finally, Experiment 4 failed to find evidence for an effect of a contextual change produced by changing the number of bottles presented at test and it appears that the rate of conditioning to AX and generalization to BX was similar after concurrent and intermixed pre-exposure. It is important to recognize that since the experiments differed in terms of several parameters, it is difficult to know exactly why Experiment 3 found an effect of the schedule but that the final experiment did not. But what it is very clear here is that in none of the four experiments, conducted with different parameters, did we find the effect previously reported: an enhancement in generalization after concurrent pre-exposure relative to the intermixed schedule.

At this point, it is important to note that only two previous studies have directly compared the effectiveness of the concurrent and intermixed pre-exposure conditions regarding the generalization of a conditioned taste aversion. One study compared the concurrent schedule with the blocked schedule (Alonso and Hall, 1999), whilst another study compared intermixed schedules with varying intervals between presentations, and whilst this interval was close to 0 in some cases (see Bennett and Mackintosh, 1999), it did not constitute the type of concurrent pre-exposure as that provided here. Other studies have also tested the effect of concurrent pre-exposure to visual stimuli with other preparations (Gibson and Walk, 1956; Wills and Mackintosh, 1999). But only the studies reported by Rodríguez and Alonso (2008) and Rodríguez et al. (2008) demonstrated greater generalization of a conditioned taste aversion after concurrent exposure in comparison with the intermixed schedule. One potentially important aspect of those experiments might be that the rats did not receive concurrent pre-exposure to two similar compounds but rather presentation of one compound and an element of such a compound (i.e., AX, X). This could be an important issue, given that evidence for stronger conditioning of the common elements of the stimuli after concurrent pre-exposure has been found only when the common elements were conditioned alone, and not when such elements were conditioned in compound with the distinctive element (see, for example, Bennett and Mackintosh, 1999). Moreover, it is important to consider that evidence for excitatory links after concurrent pre-exposure has also been found with simple flavors presented as compounds (e.g., Rescorla and Cunningham, 1978) but not when compounds of flavors were presented simultaneously (e.g., Alonso and Hall, 1999).

The results of the present study do not allow us to identify which factor might be critical in determining the results reported by Rodriguez et al. and why their findings were not replicated here. But what seems clear is that, if the incremental effect of the concurrent schedule on generalization can be obtained only in specific circumstances such as those reported by Rodriguez et al., the generality of the effect should possibly be questioned, particularly if we consider that relatively few studies have focused on the effect discussed here. This issue of generality is of critical importance for the field of perceptual learning. In particular, the findings regarding the concurrent schedule effect are precisely those in which the results of research with human and non-human animals appear to disagree. Moreover, the effects of the concurrent schedule are of particular importance for both the associative and non-associative accounts of perceptual learning.

## Author Contributions

RA completed the experiments, analyzed the data, and wrote the paper.

## Conflict of Interest Statement

The author declares that the research was conducted in the absence of any commercial or financial relationships that could be construed as a potential conflict of interest.
